# Human Parechovirus Infection, Denmark

**DOI:** 10.3201/eid2001.130569

**Published:** 2014-01

**Authors:** Thea K. Fischer, Sofie Midgley, Camilla Dalgaard, Alex Y. Nielsen

**Affiliations:** Statens Serum Institut, Copenhagen, Denmark

**Keywords:** HPeV, parechovirus, meningitis, phylogenetic, Denmark, viruses, children, infants

## Abstract

Human parechoviruses (HPeVs) often cause severe illness among young children. National surveillance with routine testing of all cerebrospinal fluid, fecal, and tissue samples was conducted during January 2009–December 2012 in all counties in Denmark (6,817 samples from 4,804 children were screened for HPeV). We detected HPeV RNA in 202 (3.0%) specimens from 149 persons. Young infants were at highest risk for HPeV, and 9 (6%) of the HPeV-infected children died, probably of their HPeV illness. HPeV3 was the most common genotype identified, and 5 closely related clades of HPeV3 circulated in Denmark throughout the study period. Our study adds perspective on the prevalence and clinical and molecular virologic characteristics of HPeV infection.

## Abstract 2

HPeV should be tested for in all young children suspected to have HPeV or enterovirus infection.

Human parechoviruses (HPeVs) have recently been recognized to cause a variety of symptoms ranging from mild diarrhea to sepsis and meningitis, particularly among young children. HPeVs belong to a large family of nonenveloped, positive-sense, single-stranded RNA viruses, the *Picornaviridae*, which comprises 12 genera (and 5 proposed genera). Six genera are associated with human infections: cardiovirus (saffold virus), cosavirus, enterovirus (EV), hepatovirus (hepatitis A), kobuvirus (Aichi virus) and HPeV. HPeV1 and HPeV2, originally known as echovirus 22 and 23 of the EV genus, respectively, were reclassified in 1999 as a separate genus (*Parechovirus*) on the basis of genetic and biologic differences (*1*). Since the reclassification, the number of known HPeVs has increased and now totals 16 genotypes (www.picornaviridae.com/parechovirus/hpev/hpev.htm).

HPeV1 is known to be associated with asymptomatic infection. HPeV3 seems to be more or less well established (*2*–*4*). The remaining known HPeVs are clinically unexplored.

Whereas HPeV3 has been reported to be associated with sepsis-like syndrome, meningitis, encephalitis, and hepatitis in neonates and young infants (*2*), most HPeV infections are asymptomatic or associated with mild respiratory and/or gastrointestinal symptoms (*4*). HPeV incidence has been reported to show a seasonal pattern in temperate climates, with different types cocirculating simultaneously (*5*). Although HPeV infections are relatively common in most settings, experience from long-term population surveillance is somewhat sparse. We used 4 years of national laboratory surveillance to describe the molecular epidemiology of HPeV, including phylogenetic characteristics of the emerging HPeV epidemic, in Denmark.

## Materials and Methods

### Study Design

During January 2009–December 2012, a total of 6,817 specimens were collected from 4,808 children from all regions of Denmark: 2,006 cerebrospinal fluid (CSF) samples from 1,952 children; 1,963 fecal samples from 1,608 children; 1,057 blood samples from 1,025 children; 682 respiratory samples from 610 children; 571 autopsy samples from 228 children; 302 swab specimens from 291 children; and 236 other samples. In general, samples were collected from patients with symptoms compatible with EV infection, particularly meningitis, sepsis-like illness, respiratory symptoms, and/or diarrhea. The CSF samples were collected from patients who typically had fever and/or clinical signs compatible with meningitis or encephalitis. The fecal samples were collected from a mixture of patients with community-acquired infection and hospitalized patients who typically had fever, diarrhea, and/or meningitis/sepsis-like illness. Autopsy material was analyzed for EV and HPeV as part of standard procedure irrespective of symptoms. Samples were sent by mail to the Department of Microbiological Diagnostics and Virology at the Statens Serum Institut (Copenhagen, Denmark) to undergo viral diagnostic and isolation and real-time PCR for EVs and HPeVs. HPeV infections detected in the same person within a 3-week period were considered to be the same infection. During the study period, the Department of Microbiological Diagnostics and Virology was the only laboratory conducting HPeV diagnostics in Denmark; therefore, the data reflect all HPeV infections detected in Denmark.

### Laboratory Analyses

#### Sample Preparation

Only fecal samples and biopsy samples needed special preparation before use of the general nucleic acid extraction protocol. Fecal samples were prepared as a 10% wt/vol suspension in minimal essential medium, followed by centrifugation at 3,500 × *g* for 30 min to remove inhibitors. Biopsy samples were suspended in Lysis/Binding Buffer from the MagNa Pure LC Total Nucleic Acid Isolation Kit (Roche Diagnostics, Mannheim, Germany), followed by homogenization.

#### Nucleic Acid Isolation

Nucleic acids were extracted from 200 μL sample material. All sample types, except CSF, were processed by using the MagNa Pure 96 DNA and Viral NA Small Volume Kit on the MagNa Pure 96 instrument (Roche Diagnostics) according to the manufacturer’s specifications. Nucleic acids from CSF were isolated by using the QIAamp DNA Blood Mini Kit on the QIAcube instrument (QIAGEN, Hilden, Germany) following the manufacturer’s specifications.

#### Amplification and Detection

For amplification, 5 μL of extracted nucleic acids were used per reverse transcription PCR (RT-PCR) reaction (total volume 25 μL) by using the OneStep RT-PCR Kit (QIAGEN). The reaction mixtures contained 1 μmol/L of each primer and 0.2 μmol/L probe. The primers and probe used have been published (*6*), and the reaction mixture also contained an assay for EV. The HPeV-specific probe was labeled with a Hex dye. The Mx3005P real-time thermocycler (Agilent Technologies A/S, Hoersholm, Denmark) was used for amplification and detection with the following settings: 50°C for 20 min, 95°C for 15 min, followed by 45 cycles of 95°C for 15 s and 55°C for 1 min.

#### Genotyping

We amplified 256–259 bp of the viral protein (VP) 3/VP1 region (from position 2159–2458 in relation to L02971) in a nested PCR. The first-round RT-PCR was conducted by using primers Harv1-F and Harv1-R (*7*) with a OneStep RT-PCR kit (QIAGEN) and the following thermocycler conditions: 50°C for 30 min, 95°C for 15 min, followed by 40 cycles of 95°C for 30 s, 42°C for 30 s, and 60°C for 45 s. The second-round PCR was conducted by using primers Harv2-F and Harv2-R (*8*) and the following thermocycler conditions: 95°C for 6 min, followed by 40 cycles of 95°C for 30 s, 60°C for 30 s, and 72°C for 45 s. This protocol was followed by a final extension at 72°C for 10 min. PCR amplification was followed by sequencing.

Before sequencing, PCR products were treated with exo-SAP IT (GE Healthcare, Buckinghamshire, UK). PCR products were sequenced by using the dideoxynucleotide chain termination method with the ABI Prism BigDye Terminator Cycle Sequencing Reaction kit on an ABI Prism 3100 automated sequencer (Applied Biosystems, Naerum, Denmark). Sequencing was performed with the forward and reverse primers from the second-round PCR. Sequences were assembled in BioNumerics 6.5 (Applied Maths, Kortrijk, Belgium). The sequences have been submitted to GenBank under accession nos. KF300772–KF300885.

### Phylogenetic Analysis

Assembled sequences were aligned with reference sequences by using the Simmonic sequence editor (www.virus-evolution.org). Phylogenetic and molecular evolutionary analyses were conducted by using MEGA 5.0 software (www.megasoftware.net). Genetic distances were calculated by using the Jukes-Cantor parameter at the nucleotide level, and the phylogenetic trees were constructed by using the maximum-likelihood method with 500 bootstrap replications (*8*).

## Results

### Sample Material

#### Study Population

During January 2009–December 2012, from 6,817 samples from 4,804 children, we detected HPeV RNA in 202 (3%) specimens from 149 (3%) children from all counties of Denmark. Of the 149 individual HPeV cases, 25 (17%) were detected in CSF samples, 105 (70%) in fecal samples, and 19 (13%) in a variety of clinical specimens (8 pharynx/tonsil swabs, 2 bronchio-lavage fluids, and 9 biopsy specimens from children who died unexpectedly [5 from abdominal lymph nodes, 1 from the small intestine, 2 from the large intestine, and 1 from pulmonary tissue]). Of the 149 individual HPeV cases, 125 had sufficient material or RNA for further VP3/VP1 regional subtyping.

#### Frequency of HPeV-infected Patients

HPeV was detected in 52 (3%) of 1,744 patients in 2009, in 31 (2%) of 1,729 patients in 2010, in 33 (2%) of 1,939 patients in 2011, and in 33 (2%) of 1,405 patients in 2012. Of the 149 HPeV-positive patients, 52 (35%) tested positive in 2009, 31 (21%) in 2010, 33 (22%) in 2011, and 33 (22%) in 2012.

**Figure 1:**
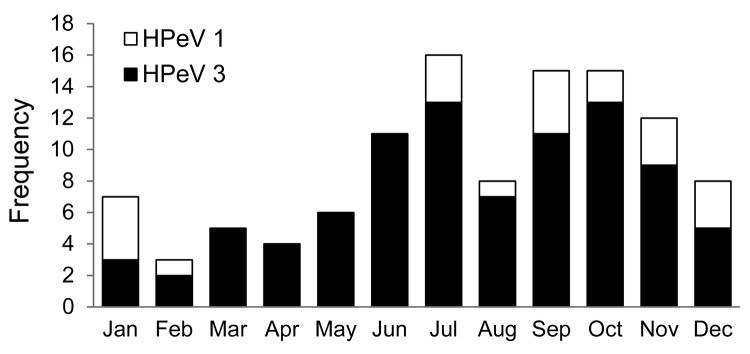
Genotype-specific seasonal distribution of laboratory-confirmed human parechovirus (HPeV) type 1 (n = 21) and HPeV3 (n = 90) in children <5 years of age, Denmark, 2009–2012.

HPeV3 infections occurred in all months of the year, albeit with a clear seasonal pattern; only few cases occurred during the winter and spring months of November–May, with a marked increase during June and July, peaking during the fall months of September and October (Figure 1). HPeV1 infections show a seasonality pattern very similar to that of the HPeV3 infections, although no cases were observed during March–June.

#### Epidemiology of HPeV-infected Persons

Age was inversely associated with risk for HPeV infection among children <5 years of age: the overall median age of HPeV-infected children was 39 days (interquartile range [IQR] 22–71 days), and the median age of HPeV-negative children was 16.3 years (IQR 318 days–41.8 years). Children with HPeV3 infection (median age 37 days [IQR 19–59 days) were significantly younger than children with HPeV1 (median age 199 days [IQR 84–303 days]).

#### Clinical Features of HPeV Infection

Information about symptoms and the source of the sample was available for 89 (60%) of children with HPeV. A wide range of symptoms were reported: slightly more cases were associated with meningitis (36 cases) than diarrhea (29) and fever (19), but sepsis-like symptoms, convulsions, apathy, and general discomfort also were reported (Table).

**Table:** Clinical characteristics of human parechovirus–infected children, Denmark, 2009–2012

Clinical characteristic	Patients, no. (%), N = 89
Fever >38.5°C	19 (21.3)*
Diarrhea and fever	29 (32.6)
Meningitis	36 (40.5)†
Sepsis-like syndrome	4 (4.5)
Sudden infant death syndrome	1 (1.1)‡

*Of the 19 patients for whom fever was the main symptom reported, 4 infants also were reported to be “irritable.” †Three of the 36 patients with meningitis also were reported to have convulsions. ‡The child who was found dead in bed (sudden unexpected infant dead), was reported to have had liquid diarrhea for 2 d before death.

### Virologic and Molecular Findings

Our sequencing of 1 of the least conservative regions of the capsid (VP3/VP1) resulted in the following: 90 HPeV3, 21 HPeV1, 8 HPeV6, 4 HPeV5, and 2 HPeV4. The genotype was assigned by BLAST analysis of the sequence against all published sequences in GenBank (http://blast.ncbi.nlm.nih.gov/).

HPeV3 was more frequently associated with disease in neonates than was any other HPeV genotype. The mean age of HPeV3-infected children was 1.6 months, compared with 8.9 months for HPeV1-infected children (n = 20), and 8.1 months for HPeV6-infected children (n = 6) (*t* test for mean age difference, p = 0.01). Sequences were available from 8 of the 9 children who died unexpectedly; of these, 4 sequences were HPeV1, 2 were HPeV3, 1 was HPeV5, and 1 was HPeV6. Of the 36 patients with reported meningitis, HPeV types were available for 32; of these, 30 (94%) types were HPeV3, and 2 (6%) were HPeV1. Of the 4 patients with sepsis-like syndrome, sample material for typing was available for 3; all 3 were identified as HPeV3. HPeV types were available for 25 of the 29 patients with reported diarrhea; of these, 19 (76%) types were HPeV3, 4 (16%) were HPeV1, and 2 (8%) were HPeV6. All 25 CSF samples were HPeV3. HPeV1 and HPeV3 were detected throughout the study period; HPeV6 was detected in all years except 2010; and HPeV5 emerged only in 2012.

**Figure 2:**
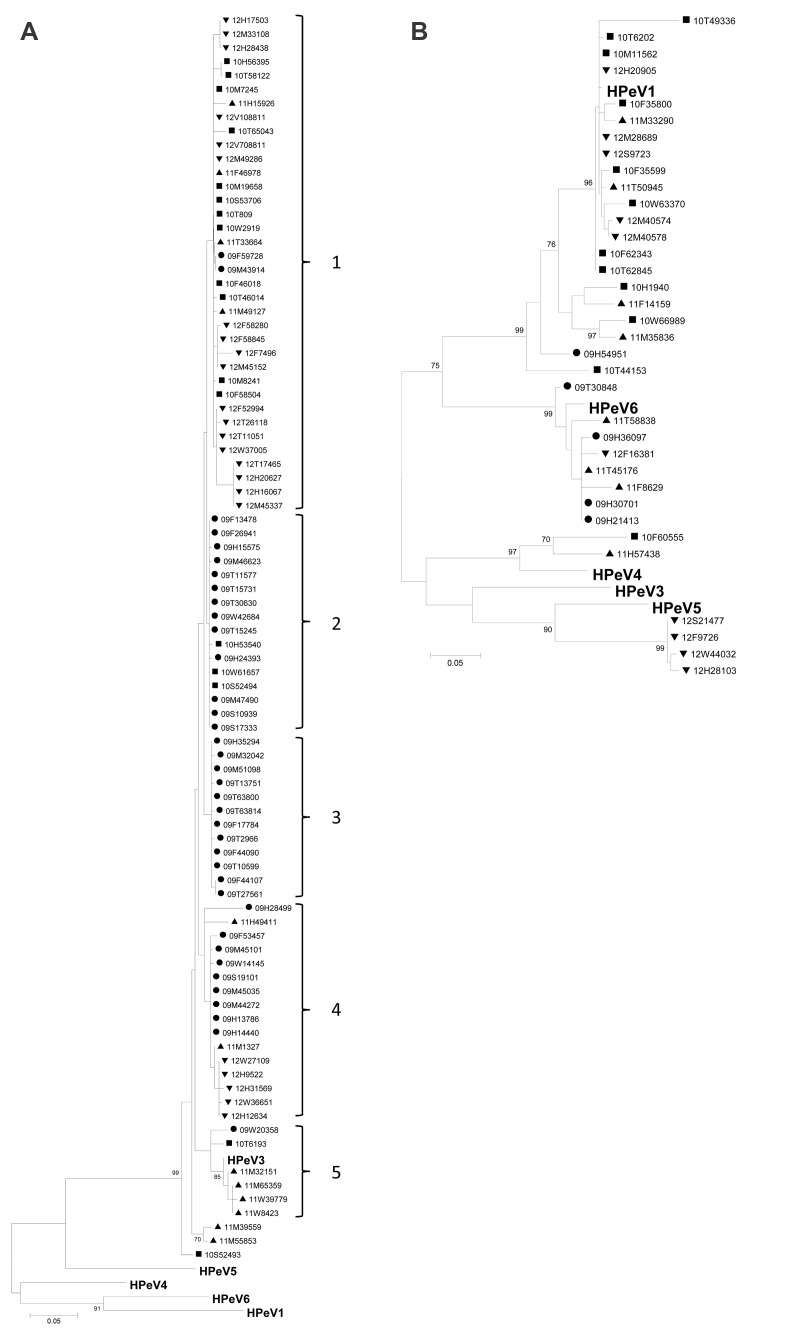
Phylogenetic analysis of the viral protein (VP) 3 (A) and VP1 (B) nucleotide sequences of human parechoviruses (HPeV), Denmark, January 2009–December 2012. Maximum-likelihood analysis of HPeVs detected in 2009 are indicated by dots, 2010 by squares, 2011 by triangles, and 2012 by inverted triangles. Reference sequences from GenBank representing the different HPeV genotypes identified in this study are shown in boldface. The following sequences were used as references: HPeV1, GenBank accession no. JX575746; HPeV3, JX826607; HPeV4, AB433629; HPeV5, JX050181; HPeV6, AB25282. Scale bars indicate nucleotide substitutions per site.

Sequence data from 124 of these 125 samples were of sufficient quality and were used to create phylogenetic trees (Figure 2, Appendix), which also included reference sequences obtained from GenBank for each genotype identified in this study. The phylogenetic analyses revealed the existence of 5 closely related clades of HPeV3 circulating in Denmark throughout the study period, with most clades found all years of the study, implying cocirculation of these clades without genetic selection of either clade in the sequenced area. In addition, there was no particular geographic distribution of the individual clades. The other genotypes appear to follow the same pattern of circulation and of little evolutionary change within each genotype over time. However, data for the genotypes other than HPeV3 were insufficient to substantiate a division into individual clades.

**Figure 3:**
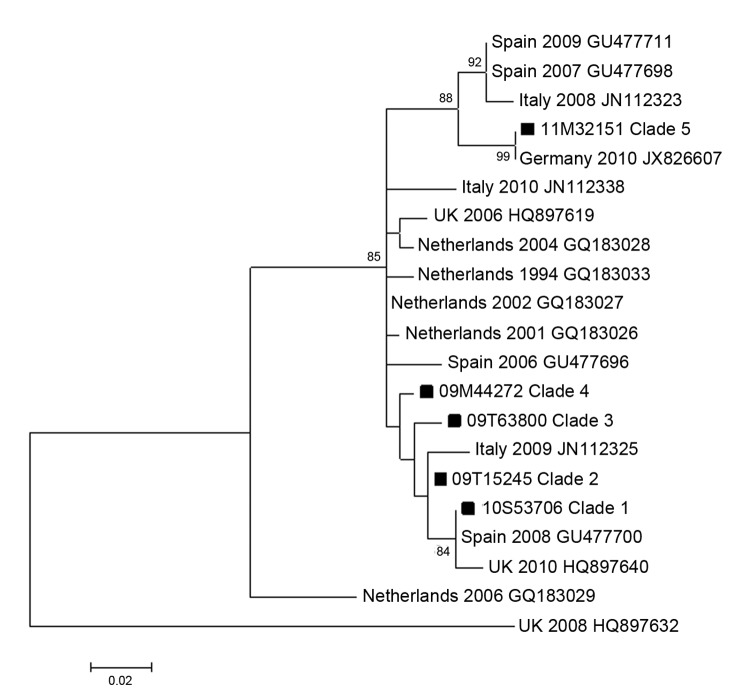
Phylogenetic analysis of strains of human parechovirus type 3 from Europe compared with a representative strain of each of the 5 clades found in Denmark (squares). Maximum-likelihood analysis with bootstrap values >70% are shown. Scale bar indicates nucleotide substitutions per site.

We created a phylogenetic tree (Figure 3) including a representative strain from each of the HPeV3 clades detected in our study in Denmark, combined with matching HPeV sequence from Europe available in GenBank. This tree showed that clades 1–4 were most closely related to strains from Spain (*9*) and Italy (*10*) during 2006–2009, whereas clade 5 was identical (in the sequenced area) to a simultaneous strain from Germany (*11*). We found an intragenotype variation of 3.9%, within HPeV1 of 5.6%, within HPeV4 of 11.6%, within HPeV5 of 1.6%, and within HPeV6 of 3.5%.

## Discussion

Our study establishes HPeV as important differential diagnosis in young and often severely ill children in Denmark with fever and/or central nervous system symptoms. The sample material received for diagnostic testing, where a significant portion of the HPeV-positive samples are CSF, illustrates the severity of these infections. Without routine testing for HPeV of infants suspected to have EV infection (EV and HPeV3 infections are indistinguishable in infants [*12*]), these children’s illnesses would not have been diagnosed, which would have resulted in unfocused treatment, such as unnecessary antimicrobial drug therapy and unnecessary long hospital stays, as suggested by Wolthers et al. (*13*). In our selected material (selected for suspected enteroviral disease), HPeV3 was by far the most prevalent HPeV type and was the only genotype detected in CSF. The high prevalence of HPeV3 contrasts with findings from other studies (*5*,*14*), which have established HPeV1 as the most common virus circulating in the population. However, these studies did not select the patients on the basis of symptoms because the children were part of healthy birth cohorts from prospective diabetes studies. In Denmark, although HPeV infections were detected throughout the year, the illness showed a marked seasonality with peaks during July and September, coinciding with the EV season. These findings underscore the need for HPeV and EV differential diagnostics in young children who have nonbacterial meningitis and severe disease in general caused by the similar clinical characteristics of HPeV and EV (*12*). The observed seasonality (i.e., occurrence of HPeV3 every year) indicates an endemic pattern of HPeV infection in Denmark. This finding contrasts with other HPeV studies from the United Kingdom and the Netherlands, where a biannual cycle of HPeV3 infections has been observed; the reason for this difference is unknown (*15*,*16*).

In our study, the children infected with HPeV (and in particular HPeV3) were very young (median age 39 days), which is consistent with prior studies (*17*,*18*). However, a recent study from Japan (*5*) has established that adults also can be infected with HPeV3, possibly resulting in epidemic myalgia.

We also showed that HPeV3 in Denmark did not undergo significant genetic diversification during 2009–2012, at least not in the sequenced part of the capsid (VP3/VP1) (Figure 2, Appendix). This observation was in line with comparisons of the strains from Denmark with strains from Europe in which only limited variation periodically (1994–2012) and geographically was observed (Figure 3). The genetic distances in the trees are small (which further substantiates the results of limited evolutionary change). These indications of limited genetic evolution correspond well with results from a study in the Netherlands that described HPeV during 2000–2007 (*19*). The reason for the lack of intratypic diversification of the otherwise intertypically heterogenic capsid area is unknown. Perhaps most infections are subclinical and do not elicit an immunologic response, which might otherwise cause the virus to evolve to escape the immune response. Structural constraints might also exist that limit the genetic variation to ensure proper capsid formation.

Our data supported previous findings that HPeV3 is commonly associated with meningitis/sepsis symptoms among young infants, and we found that convulsions and apathy were reported among children with such severe cases. Because HPeV3 has been reported to cause white matter injury in 9 of 10 infected neonates, resulting in a range of complications from cerebral palsy in 1 child to epilepsy in another child to learning disabilities in a third child (*20*), focused longitudinal follow-up and cognitive evaluation of these children during childhood might be indicated to better understand the long-term consequences of this emerging viral disease.

In conclusion, HPeV was a clinically significant virus rivaling EVs in young children in Denmark. In particular, HPeV3 was circulating in our clinically selected material, with 5 different clades cocirculating over most of the years of the study. The patients were from all around Denmark demonstrating the need for central or disseminated HPeV diagnostics in Denmark so that HPeV is tested for in all young children suspected of infection with HPeV or EV. Testing might possibly reduce the length of hospitalization and limit the inappropriate use of antimicrobial drugs. Furthermore, doctors and especially pediatricians should be aware of the symptoms of HPeV-associated diseases and should be encouraged to collect a sample (primarily CSF and preferably combined with a fecal sample) for diagnostics. Centralized surveillance of this virus could provide deeper insight into the behavior of HPeV and might shed light on the clinical significance of the HPeVs other than type 3.

## References

[1] Stanway G, Hyypia T. Parechoviruses. J Virol. 1999;73:5249–54 . 10364270 10.1128/jvi.73.7.5249-5254.1999 PMC112579

[2] Boivin G, Abed Y, Boucher FD. Human parechovirus 3 and neonatal infections. Emerg Infect Dis. 2005;11:103–5. 10.3201/eid1101.040606 15705330 PMC3294360

[3] Mizuta K, Kuroda M, Kurimura M, Yahata Y, Sekizuka T, Aoki Y, Epidemic myalgia in adults associated with human parechovirus type 3 infection, Yamagata, Japan, 2008. Emerg Infect Dis. 2012;18:1787–93. 10.3201/eid1811.111570 23095469 PMC3559140

[4] Stanway G, Joki-Korpela P, Hyypia T. Human parechoviruses—biology and clinical significance. Rev Med Virol. 2000;10:57–69. 10.1002/(SICI)1099-1654(200001/02)10:1<57::AID-RMV266>3.0.CO;2-H 10654005

[5] Kolehmainen P, Oikarinen S, Koskiniemi M, Simell O, Ilonen J, Knip M, Human parechoviruses are frequently detected in stool of healthy Finnish children. J Clin Virol. 2012;54:156–61. 10.1016/j.jcv.2012.02.006 22406272

[6] Baumgarte S, de Souza Luna LK, Grywna K, Panning M, Drexler JF, Karsten C, Prevalence, types, and RNA concentrations of human parechoviruses, including a sixth parechovirus type, in stool samples from patients with acute enteritis. J Clin Microbiol. 2008;46:242–8. 10.1128/JCM.01468-07 18057123 PMC2224249

[7] Harvala H, Robertson I, McWilliam Leitch EC, Benschop K, Wolthers KC, Templeton K, Epidemiology and clinical associations of human parechovirus respiratory infections. J Clin Microbiol. 2008;46:3446–53. 10.1128/JCM.01207-08 18753351 PMC2566073

[8] Tamura K, Peterson D, Peterson N, Stecher G, Nei M, Kumar S. MEGA5: molecular evolutionary genetics analysis using maximum likelihood, evolutionary distance, and maximum parsimony methods. Mol Biol Evol. 2011;28:2731–9. 10.1093/molbev/msr121 21546353 PMC3203626

[9] Piñeiro L, Vicente D, Montes M, Hernández-Dorronsoro U, Cilla G. Human parechoviruses in infants with systemic infection. J Med Virol. 2010;82:1790–6. 10.1002/jmv.21878 20827778

[10] Piralla A, Furione M, Rovida F, Marchi A, Stronati M, Gerna G, Human parechovirus infections in patients admitted to hospital in northern Italy, 2008–2010. J Med Virol. 2012;84:686–90. 10.1002/jmv.23197 22337310 PMC7166678

[11] Eis-Hübinger AM, Eckerle I, Helmer A, Reber U, Dresbach T, Buderus S, Two cases of sepsis-like illness in infants caused by human parechovirus traced back to elder siblings with mild gastroenteritis and respiratory symptoms. J Clin Microbiol. 2013;51:715–8. 10.1128/JCM.02731-12 23241372 PMC3553862

[12] Verboon-Maciolek MA, Krediet TG, Gerards LJ, de Vries LS, Groenendaal F, van Loon AM. Severe neonatal parechovirus infection and similarity with enterovirus infection. Pediatr Infect Dis J. 2008;27:241–5. 10.1097/INF.0b013e31815c1b07 18277927

[13] Wolthers KC, Benschop KS, Schinkel J, Molenkamp R, Bergevoet RM, Spijkerman IJ, Human parechoviruses as an important viral cause of sepsislike illness and meningitis in young children. Clin Infect Dis. 2008;47:358–63. 10.1086/589752 18558876

[14] Tapia G, Cinek O, Witso E, Kulich M, Rasmussen T, Grinde B, Longitudinal observation of parechovirus in stool samples from Norwegian infants. J Med Virol. 2008;80:1835–42. 10.1002/jmv.21283 18712841

[15] Harvala H, Robertson I, Chieochansin T, McWilliam Leitch EC, Templeton K, Simmonds P. Specific association of human parechovirus type 3 with sepsis and fever in young infants, as identified by direct typing of cerebrospinal fluid samples. J Infect Dis. 2009;199:1753–60. 10.1086/599094 19456229

[16] Harvala H, Wolthers KC, Simmonds P. Parechoviruses in children: understanding a new infection. Curr Opin Infect Dis. 2010;23:224–30. 10.1097/QCO.0b013e32833890ca 20414971

[17] Benschop KS, Schinkel J, Minnaar RP, Pajkrt D, Spanjerberg L, Kraakman HC, Human parechovirus infections in Dutch children and the association between serotype and disease severity. Clin Infect Dis. 2006;42:204–10. 10.1086/498905 16355330

[18] Selvarangan R, Nzabi M, Selvaraju SB, Ketter P, Carpenter C, Harrison CJ. Human parechovirus 3 causing sepsis-like illness in children from midwestern United States. Pediatr Infect Dis J. 2011;30:238–42. 10.1097/INF.0b013e3181fbefc8 20948454

[19] van der Sanden S. de BE, Vennema H, Swanink C, Koopmans M, van der Avoort H. Prevalence of human parechovirus in the Netherlands in 2000 to 2007. J Clin Microbiol. 2008;46:2884–9. 10.1128/JCM.00168-08 18614653 PMC2546772

[20] Verboon-Maciolek MA, Groenendaal F, Hahn CD, Hellmann J, van Loon AM, Boivin G, Human parechovirus causes encephalitis with white matter injury in neonates. Ann Neurol. 2008;64:266–73. 10.1002/ana.21445 18825694

